# A microbiota‐based predictive model for type 2 diabetes remission induced by dietary intervention: From the CORDIOPREV study

**DOI:** 10.1002/ctm2.326

**Published:** 2021-04-06

**Authors:** Cristina Vals‐Delgado, Juan F. Alcala‐Diaz, Irene Roncero‐Ramos, Ana Leon‐Acuña, Helena Molina‐Abril, Francisco M. Gutierrez‐Mariscal, Juan L. Romero‐Cabrera, Silvia de la Cruz‐Ares, Ben van Ommen, Justo P. Castaño, Jose M. Ordovas, Pablo Perez‐Martinez, Javier Delgado‐Lista, Antonio Camargo, Jose Lopez‐Miranda

**Affiliations:** ^1^ Lipids and Atherosclerosis Unit Internal Medicine Unit Reina Sofia University Hospital Cordoba Spain; ^2^ Department of Medicine University of Cordoba Cordoba Spain; ^3^ Maimonides Biomedical Research Institute of Cordoba (IMIBIC) Cordoba Spain; ^4^ CIBER Fisiopatologia de la Obesidad y Nutrición (CIBEROBN) Instituto de Salud Carlos III Cordoba Spain; ^5^ Department of Applied Mathematics I University of Seville Seville Spain; ^6^ Netherlands Institute for Applied Science (TNO) Research Group Microbiology & Systems Biology Zeist The Netherlands; ^7^ Department of Cell Biology Physiology and Immunology University of Cordoba Córdoba Spain; ^8^ Nutrition and Genomics Laboratory J.M.‐US Department of Agriculture Human Nutrition Research Center on Aging at Tufts University Boston Massachusetts USA; ^9^ IMDEA Alimentacion Madrid, Spain. CNIC Madrid Spain

Dear Editor,

Type 2 diabetes is widely considered as a chronic, progressive disease that is a consequence of the seemingly inexorable decline in β‐cell function.^1^ However, recent studies have demonstrated that in the early stages of development, it may be reversible. Indeed, in this regard, primary support derived from patients undergoing bariatric surgery has provided the earliest evidence of type 2 diabetes remission. In fact, the normalization of plasma glucose levels can occur in some patients just days after bariatric surgical intervention, and even before achieving significant weight loss.^2^ This observation points to a relevant glucoregulatory role of the gastrointestinal tract. It has been proposed that a number of different, but not mutually exclusive, potential mechanisms may contribute toward this effect, including changes in bile acid metabolism, gastrointestinal tract nutrient sensing and glucose utilization, incretins, and gut microbiota.^3^

Recent studies have shown that it is possible to induce type 2 diabetes remission by weight loss with calorie restriction interventions.^4^ Depletion of the gut microbiota with antibiotic treatment and fecal microbiota transplantation suggest that the gut microbiota plays a causal role in the beneficial effects of calorie restriction, especially by lowering body weight and hepatic lipid accumulation.^5^ Furthermore, it has been observed that calorie restriction and diabetes remission are associated with an improvement of gut permeability and a reduction in inflammatory and endotoxemia biomarkers.^6^, ^7^

Thus, it is noteworthy that the two approaches that, to date, are known to enable type 2 diabetes remission have plausibly suggested that a role is played by the gut microbiota. This idea is further strengthened by the reported association between this disease and the gut microbiota. Alterations in the gut microbiota of patients with type 2 diabetes have been described,^8^ which adds to the potential causal relationship between the gut microbiome and impaired glucose metabolism, a notion which is supported by studies based on fecal transfer in patients with metabolic syndrome.^9^

Lifestyle modifications, including the implementation of healthy diets, have been shown to have a beneficial effect on type 2 diabetes prevention.^10^ In particular, it has been suggested that the impact of dietary intervention on metabolism is associated with baseline gut microbiota composition. Hence, microbiome biomarkers could potentially be used to identify subjects who might benefit from specific dietary interventions.^11^

Our study, conducted in 110 newly diagnosed type 2 diabetes patients with coronary heart disease (CHD) within the Coronary Diet Intervention with Olive Oil and Cardiovascular Prevention (CORDIOPREV) study, evaluated whether baseline gut microbiota composition, in addition to the classic type 2 diabetes risk‐associated variables, improves the identification of patients who underwent type 2 diabetes remission achieved by two dietary models (low‐fat or Mediterranean diet) after a 5‐year follow‐up (responders, *n* = 44) and those who did not respond to the dietary intervention (nonresponders, *n* = 66), with both groups presenting the same adherence to both diets, and without promoting changes in lifestyle such as weight loss or medication. We observed a higher weight, body mass index, waist circumference and glucose levels, and a lower disposition index at baseline in the nonresponders than in the responders (*p* < 0.05) (Tables 1 and 2).

**TABLE 1:** Baseline characteristics of the newly diagnosed type 2 diabetes population for type 2 diabetes mellitus remission study and of the population with analyzed gut microbiota

	Responders	Nonresponders		Responders^†^	Nonresponders^†^	
	(*n* = 73)	(*n* = 110)	*p* value	(*n* = 44)	(*n* = 66)	*p* value
Men/women	60/13	92/18	0.799	36/8	56/10	0.674
Age (years)	60.8 ± 1.0	59.3 ± 0.9	0.252	60.1 ± 1.3	57.7 ± 1.2	0.198
Weight (kg)	80.2 ± 1.3	88.4 ± 1.4	<0.001	78.2 ± 1.7	88.0 ± 1.8	<0.001
Body mass index (kg/m^2^)	29.9 ± 0.4	32.1 ± 0.4	0.001	29.2 ± 0.6	31.7 ± 0.5	0.002
Waist circumference (cm)	101 ± 1	108 ± 1	<0.001	99 ± 1.3	108 ± 1.3	<0.001
Systolic blood pressure (mm Hg)	137 ± 3	138 ± 2	0.717	137 ± 3	135 ± 2	0.518
Diastolic blood pressure (mm Hg)	76.6 ± 1.6	77.1 ± 1.0	0.755	76.4 ± 2.1	76.5 ± 1.4	0.945
Triglycerides (mmol/L)	1.48 ± 0.10	1.69 ± 0.07	0.090	1.60 ± 0.15	1.71 ± 0.09	0.478
Total cholesterol (mmol/L)	4.16 ± 0.08	4.31 ± 0.08	0.203	4.24 ± 0.11	4.29 ± 0.11	0.726
HDL‐cholesterol (mmol/L)	1.11 ± 0.03	1.06 ± 0.02	0.141	1.12 ± 0.05	1.04 ± 0.03	0.116
LDL cholesterol (mmol/L)	2.31 ± 0.07	2.42 ± 0.07	0.302	2.32 ± 0.10	2.35 ± 0.09	0.828
C‐reactive protein (nmol/L)	37.1 ± 5.3	33.5 ± 3.6	0.558	37.8 ± 6.8	32.2 ± 4.5	0.480
HbA1c (mmol/mol)	47.8 ± 0.9	50.7 ± 0.9	0.032	48.1 ± 1.2	50.7 ± 1.3	0.176
HbA1c (%)	6.53 ± 0.08	6.79 ± 0.08	0.032	6.55 ± 0.11	6.79 ± 0.12	0.176
Glucose (mmol/L)	5.50 ± 0.09	6.58 ± 0.14	<0.001	5.59 ± 0.12	6.47 ± 0.20	0.001
Insulin (nmol/L)	64.4 ± 5.5	93.3 ± 7.8	0.007	64.8 ± 7.1	94.8 ± 11.9	0.058
HOMA‐IR	3.49 ± 0.42	4.84 ± 0.32	0.010	3.93 ± 0.65	4.76 ± 0.45	0.281
Insulin sensitivity index	3.16 ± 0.20	2.37 ± 0.12	<0.001	3.02 ± 0.26	2.47 ± 0.16	0.063
Insulinogenic index	0.70 ± 0.19	0.68 ± 0.14	0.921	0.89 ± 0.26	0.80 ± 0.22	0.795
Hepatic insulin resistance index	1421 ± 168	1970 ± 129	0.009	1616 ± 263	1943 ± 182	0.293
Muscle insulin sensitivity index (×10^2^)	1.93 ± 0.22	2.20 ± 0.25	0.452	1.74 ± 0.29	2.23 ± 0.35	0.313
Disposition index	0.68 ± 0.06	0.43 ± 0.02	<0.001	0.65 ± 0.05	0.46 ± 0.03	0.002
Smoking (%)	11.4	16.7	0.440	12.3	11.8	0.917
Hypertension (%)	57.5	65.5	0.279	61.4	60.6	0.936
History of peripheral vascular disease (%)	0.0	4.5	0.065	0.0	6.1	0.096
History of stroke or TIA (%)	4.1	4.5	0.888	4.5	4.5	1.000
History of myocardial infarction (%)	53.4	55.5	0.787	56.8	57.6	0.937
History of anginá (%)	40.0	39.7	0.970	38.6	36.4	0.809
History of PCI (%)	93.2	98.2	0.082	90.9	98.5	0.062
History of CABG (%)	5.5	2.7	0.342	6.8	1.5	0.146
**Baseline medication (%)**						
Anti‐aggregates	94.5	96.4	0.550	95.5	100	0.080
Beta‐blockers	57.5	65.5	0.279	56.8	68.2	0.225
ACE inhibitors	13.7	17.3	0.517	13.6	12.1	0.815
Diuretics	39.7	41.8	0.778	29.5	37.9	0.368
Angiotensin‐II receptor blockers (ARBs)	20.5	23.6	0.624	22.7	19.7	0.702
Calcium antagonists	11.0	19.1	0.140	13.6	19.7	0.410
Nitrates	15.1	7.3	0.090	11.4	9.1	0.697
Anti‐arrhythmics	2.7	0.9	0.340	2.3	1.5	0.771
Oral anticoagulants	1.4	0.9	0.769	0.0	1.5	0.412
Statins	86.3	88.2	0.707	95.5	95.5	1.000
Other hypolipidemics	6.8	7.3	0.913	6.8	6.1	0.873
Proton pump inhibitors	83.6	82.7	0.883	86.4	89.4	0.630
Tranquilizers	6.8	8.2	0.740	9.1	10.6	0.795

Abbreviations: CABG, coronary artery bypass grafting; PCI, percutaneous coronary intervention; TIA, transient ischemic attack.

Our study was conducted in 183 newly diagnosed type 2 diabetes patients, 110 from which had available feces samples and had not received antibiotic treatment within 3 months before sample collection. Data are mean ± SEM. Responders group: patients who reverted from type 2 diabetes after 5 years of dietary intervention follow‐up. Nonresponders group: patients who remained with type 2 diabetes after 5 years of follow‐up. Responders^†^: patients who reverted from type 2 diabetes after 5 years of dietary intervention follow‐up to which we have the availability of fecal sample. Nonresponders^†^: patients who remained with type 2 diabetes after 5 years of follow‐up to which we have the availability of fecal sample. *p*‐values were calculated by one‐way ANOVA. Gender *p*‐value by chi‐square analysis.

**TABLE 2:** Baseline characteristics of the newly diagnosed type 2 diabetes population for type 2 diabetes mellitus remission study compared with the population with analyzed gut microbiota

	Responders	Responders^†^		Nonresponders	Nonresponders^†^	
	(*n* = 73)	(*n* = 44)	*p* value	(*n* = 110)	(*n* = 66)	*p* value
Men/women	60/13	36/8	0.959	92/18	56/10	0.831
Age (years)	60.8 ± 1.0	60.1 ± 1.3	0.649	59.3 ± 0.9	57.7 ± 1.2	0.304
Weight (kg)	80.2 ± 1.3	78.2 ± 1.7	0.341	88.4 ± 1.4	88.0 ± 1.8	0.835
Body mass index (kg/m^2^)	29.9 ± 0.4	29.2 ± 0.6	0.361	32.1 ± 0.4	31.7 ± 0.5	0.639
Waist circumference (cm)	101 ± 1	99 ± 1	0.304	108 ± 1	108 ± 1	0.725
Triglycerides (mmol/L)	1.48 ± 0.10	1.60 ± 0.15	0.515	1.69 ± 0.07	1.71 ± 0.09	0.868
Total cholesterol (mmol/L)	4.16 ± 0.08	4.24 ± 0.11	0.543	4.31 ± 0.08	4.29 ± 0.11	0.895
HDL cholesterol (mmol/L)	1.11 ± 0.03	1.12 ± 0.05	0.838	1.06 ± 0.2	1.04 ± 0.03	0.667
LDL cholesterol (mmol/L)	2.31 ± 0.07	2.32 ± 0.10	0.916	2.42 ± 0.07	2.35 ± 0.09	0.570
C‐reactive protein (nmol/L)	37.1 ± 5.3	37.8 ± 6.8	0.938	33.5 ± 3.6	32.2 ± 4.5	0.832
HbA1c (mmol/mol)	47.8 ± 0.9	48.1 ± 1.2	0.870	50.7 ± 0.9	50.7 ± 1.3	0.992
HbA1c (%)	6.53 ± 0.08	6.55 ± 0.11	0.870	6.79 ± 0.08	6.79 ± 0.12	0.992
Glucose (mmol/L)	5.50 ± 0.09	5.59 ± 0.12	0.561	6.58 ± 0.14	6.47 ± 0.20	0.649
Insulin (nmol/L)	64.4 ± 5.5	64.8 ± 7.1	0.968	93.3 ± 7.8	94.8 ± 11.9	0.912
HOMA‐IR	3.49 ± 0.42	3.93 ± 0.65	0.555	4.84 ± 0.32	4.76 ± 0.45	0.886
Insulin sensitivity index	3.16 ± 0.20	3.02 ± 0.26	0.666	2.37 ± 0.12	2.47 ± 0.16	0.594
Insulinogenic index	0.70 ± 0.19	0.89 ± 0.26	0.549	0.68 ± 0.14	0.80 ± 0.22	0.621
Hepatic insulin resistance index	1421 ± 168	1616 ± 263	0.512	1970 ± 129	1943 ± 182	0.904
Muscle insulin sensitivity index (×10^2^)	1.93 ± 0.22	1.74 ± 0.29	0.583	2.20 ± 0.25	2.23 ± 0.35	0.920
Disposition index	0.68 ± 0.06	0.65 ± 0.05	0.713	0.43 ± 0.02	0.46 ± 0.03	0.940

Our study was conducted in 183 newly diagnosed type 2 diabetes patients, 110 from which had available feces samples and had not received antibiotic treatment within 3 months before sample collection. Data are mean ± SEM. Responders: patients who reverted from type 2 diabetes after 5 years of dietary intervention follow‐up. Responders^†^: patients who reverted from type 2 diabetes after 5 years of dietary intervention follow‐up to which we have availability of fecal sample. Nonresponders: patients who remained with type 2 diabetes after 5 years of follow‐up. Nonresponders^†^: patients who remained with type 2 diabetes after 5 years of follow‐up to which we have availability of fecal sample. *p*‐Values were calculated by one‐way ANOVA. Gender *p* value by chi‐square analysis. Significant differences (*p *< 0.05).

First of all, Linear discriminant analysis Effect Size (LEfSe) (Figure 1) showed that gut microbiota in the responders group was characterized by the *Ruminococcus* genus of the *Lachnospiraceae* family. In contrast, the baseline gut microbiota in the nonresponders group was enriched in the *Porphyromonadaceae* family and *Parabacteroides* genus. However, the bacterial richness and diversity assessed by the main α diversity indexes were similar between groups, and no significant differences were found.

**FIGURE 1:**
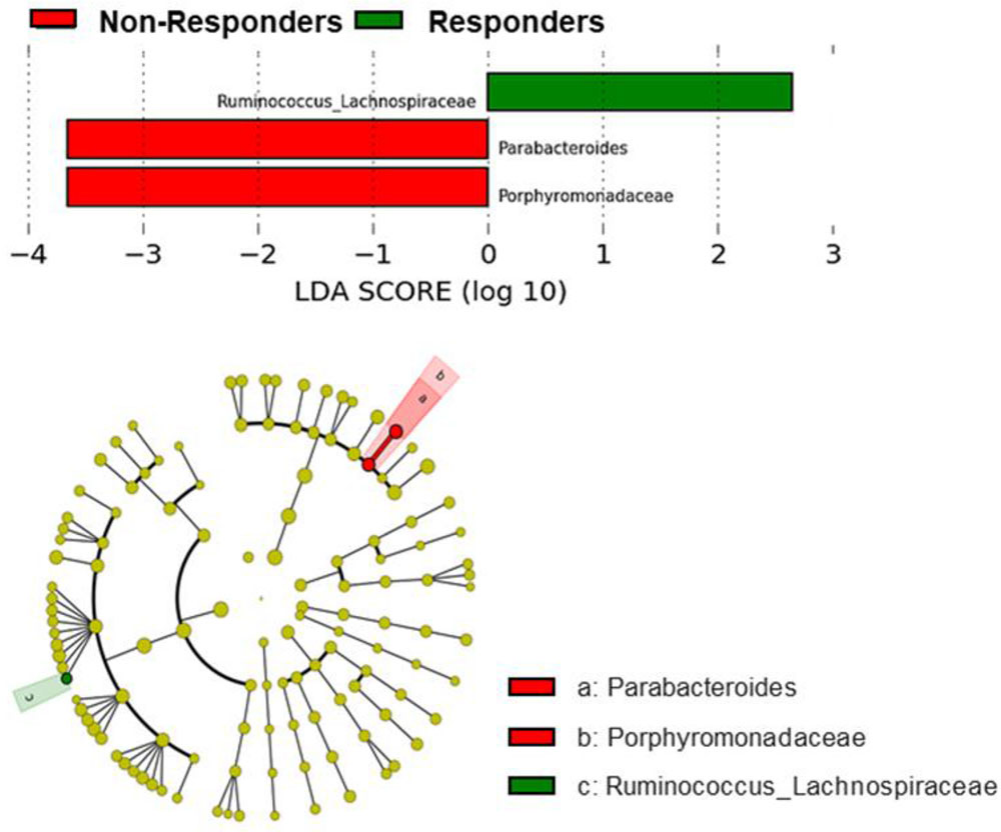
Differently abundant taxa identified using Linear discriminant analysis Effect Size (LEfSe) analysis. The most differently abundant taxa between the groups of study are represented in a bar graph according to the LDA score (log 10) and in a taxonomic cladogram. Only taxa with *p *< 0.05 and an LDA score significant threshold | > 2| are shown. The responders group (green color) was characterized by the *Ruminococcus* genus of the *Lachnospiraceae* family. Nonresponders group was enriched in the *Porphyromonadaceae* family and *Parabacteroides* genus (red color). In the taxonomic cladogram, each successive circle represents a different phylogenetic level. The order (from the center outwards) is phylum, class, family, and genus levels. Differing taxa are listed on the right‐hand side of the cladogram

Emerging evidence suggests that the host's metabolic response to a nutritional or dietary intervention depends on microbiome composition. In fact, a recent study showed that the gut microbiota, together with clinical, anthropometric and lifestyle data, enables us to make an accurate prediction of the postprandial glucose individual response to different foods.^11^ Moreover, this prediction was demonstrated to be useful for designing personalized dietary interventions aimed at reducing postprandial glucose.^11^ In order to evaluate the potential of gut microbiota composition as a predictive factor of type 2 diabetes remission, we built several random forest classifier models, which were evaluated using 10‐fold cross‐validation method. These analyses showed that the addition of the microbiome to the classic variables associated with diabetes risk improved our ability to differentiate between those responder individuals who would benefit from the consumption of two dietary models (low‐fat or Mediterranean diet) and those whose diabetes would remain despite the dietary intervention. These models had a sensitivity of 83% and specificity of 66% (Figure 2A) and a sensitivity of 78% and specificity of 68% (Figure S1), respectively.

**FIGURE 2:**
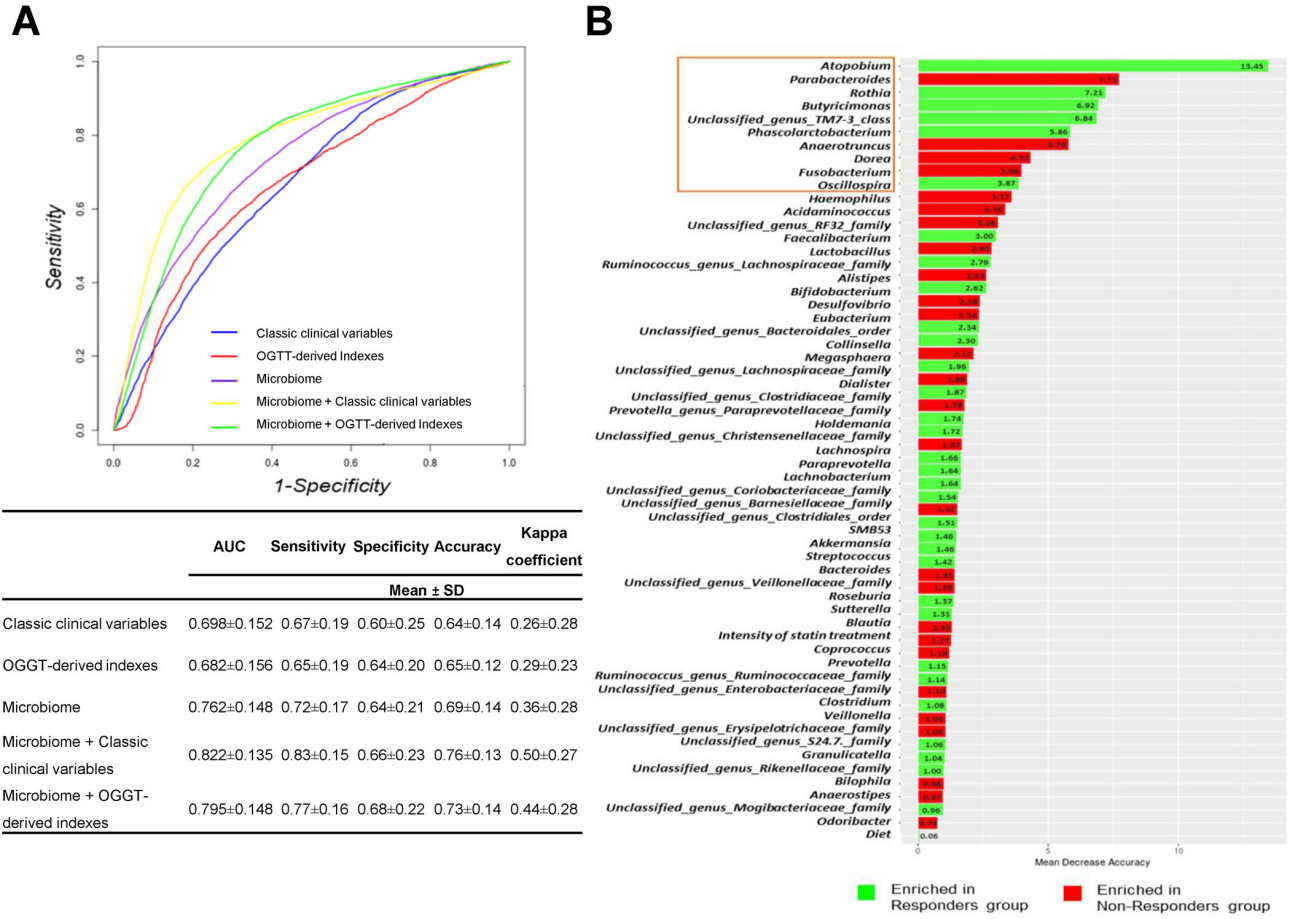
Random forest predictive models for diet consumption‐induced type 2 diabetes remission. (A) Multivariate Receiver operating characteristic (ROC) curve analysis based on the random forest classification models built. Data are mean ± standard deviation (SD) and ROC curves are an average of the predictive models using a cross‐validation method. The classic variables were the clinical parameters known to be risk factors for type 2 diabetes development: gender, age, body mass index, high‐density lipoprotein (HDL), triglycerides, glycosylated hemoglobin, type A1C (HbA1c), physical activity, dietary consumption of fruit and vegetables, use of antihypertensive medication, and family history of diabetes; oral glucose tolerance test (OGTT)‐derived indexes: homeostatic model assessment of insulin resistance, disposition index, hepatic insulin resistance index, insulin sensitivity index, muscle insulin sensitivity index, and insulinogenic index. The models were adjusted by the diet consumed (low‐fat or Mediterranean diet) and intensity of statin treatment including these variables in all the models. AUC, the area under the ROC curve. (B) Variable importance values of the microbiome model, which included all the genera present in the patients’ gut microbiota. The variable importance values are represented by the mean of the decrease in accuracy of the models when these taxa are removed. A higher mean decrease in accuracy or bar lengths indicates the greater importance of the variable. The top 10 most discriminant genera are highlighted with a square

We also evaluated the putative role of the microbiome in evaluating the probability of dietary consumption‐induced type 2 diabetes remission by COX regressions analysis. To do this, we built a response prediction score for patients based on their microbiome profile, using the 10 bacterial taxa with the highest importance in the predictive microbiome‐based model, as assessed by mean decrease accuracy (MDA, Figure 2B). We evaluated the probability of type 2 diabetes remission by COX regressions of the score generated by categorizing patients into (ascending) tertiles of the microbiome‐based response prediction score value (Figure 3A).

**FIGURE 3:**
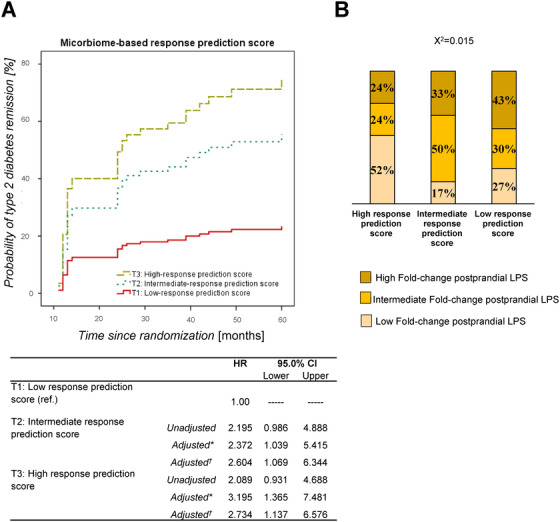
Type 2 diabetes remission according to the microbiome score. (A) Probability of type 2 diabetes remission by the dietary intervention (consumption of low‐fat or Mediterranean diet) by COX regression according to the microbiome‐based response prediction score. The microbiome‐based response prediction score was built by adding up the mean decrease accuracy for the 10 most discriminant bacterial taxa, with a positive or negative value, according to the above protective or risk tertiles and disregarding the tertiles with neutral effect (added as 0). The detrimental or beneficial role was determined by observing a higher mean baseline abundance in nonresponders or responders, respectively. In this way, for a detrimental genus: tertile 1 was scored as 1 (protective), tertile 2 as 0 (neutral effect), and tertile 3 as ‐1 (risk), and the opposite for a beneficial genus: tertile 1 as ‐1 (risk), tertile 2 as 0 (neutral effect), and tertile 3 as 1 (protective). The data represent the response prediction score values by tertiles (ascending), T1: low‐response prediction score (solid line); T2: intermediate‐response prediction score (short dash); T3: high‐response prediction score (long dash). *The model was adjusted by age, body mass index, gender, diet, high‐density lipoprotein (HDL), triglycerides, and intensity of statin treatment. †This model was adjusted by the above variables as well as by smoking, history of myocardial infarction, history of stroke or transient ischemic attack, history of peripheral vascular disease, hypertension, history of coronary artery bypass grafting, history of percutaneous coronary intervention. (B) Relationship between microbiome‐based response prediction score and postprandial increase of lipopolysaccharide (LPS) plasma levels. The relationship between the groups defined according to the microbiome‐based response prediction score by ascending tertiles and the tertiles of the postprandial fold change of LPS plasma levels was analyzed using chi‐square analysis

A large number of trials have demonstrated the effectiveness of weight loss or carbohydrate restriction diets, based on a change in macronutrients, to induce diabetes remission,^12^ in which the ability of β‐cells to recover glucose‐stimulated insulin secretion seems to be crucial.^4^ This suggests that the microbiota profile is associated with β‐cell functionality, as evidenced by the lack of baseline differences between responders and nonresponders when patients were classified by ascending tertiles, with the exception of the disposition index (Table S4).

In addition, we evaluated the relationship between the groups defined according to their microbiome‐based response prediction score by ascending tertiles, with the tertiles of the postprandial fold‐change of endotoxin lipopolysaccharide (LPS) plasma levels using the chi‐square test. An inverse relationship was observed between the response prediction score and the postprandial increase of LPS. Thus, in the group of patients categorized as high‐response prediction score, we found a higher percentage of patients with a low postprandial increase in LPS. The intermediate‐response prediction score group was characterized by a higher percentage of patients with an intermediate postprandial increase in LPS and the low‐response prediction score group was composed of a higher percentage of patients with a high postprandial increase in LPS (*χ* ^2^ = 0.015) (Figure 3B).

Our study has certain limitations, such as the specific geographical area to which the population belongs, and the fact that type 2 diabetes remission was not the primary endpoint of the CORDIOPREV trial, which includes patients with CHD, thus limiting our findings to individuals with this comorbidity.

In conclusion, our results suggest that there is a gut microbiota profile associated with type 2 diabetes remission and provide compelling evidence of a potential role of the microbiome as a predictive factor for responders to diet‐induced type 2 diabetes remission in newly diagnosed patients with CHD. Further studies are needed to assess gut microbiota–diet interaction in the host metabolism response and to understand the exact impact of this relationship on type 2 diabetes remission.

## CONFLICT OF INTEREST

The authors declare that there is no conflict of interest.

## ETHICS APPROVAL AND CONSENT TO PARTICIPATE

The trial protocol and all amendments were approved by the Reina Sofia University Hospital Ethics Committee, following the Helsinki declaration and good clinical practices. All patients signed an informed consent to participate in the study.

## AUTHOR CONTRIBUTIONS

Cristina Vals‐Delgado and Antonio Camargo wrote the draft manuscript. Juan F. Alcala‐Diaz and Irene Roncero‐Ramos collected data and performed the classification of participants Cristina Vals‐Delgado, Irene Roncero‐Ramos, Francisco M. Gutierrez‐Mariscal, Silvia de la Cruz‐Ares performed the experiments. Juan F. Alcala‐Diaz, Ana Leon‐Acuña, and Juan L. Romero‐Cabrera performed the medical revisions of participants and clinical databases. Cristina Vals‐Delgado and Helena Molina‐Abril performed the statistical analysis. Cristina Vals‐Delgado, Antonio Camargo, and Jose Lopez‐Miranda interpreted the data and contributed to the discussion. Antonio Camargo, Irene Roncero‐Ramos, Ben van Ommen, Justo P. Castaño, Jose M. Ordovas, Pablo Perez‐Martinez, Javier Delgado‐Lista, and Jose Lopez‐Miranda contributed to the writing of the manuscript and revised it critically for important intellectual content. Antonio Camargo and Jose Lopez‐Miranda are the guarantors of this work and, as such, had full access to all the data in the study and take responsibility for the integrity of the data and the accuracy of the data analysis.

## DATA AVAILABILITY STATEMENT

The sequences obtained in this study are open available in the NCBI Sequence Read Archive (SRA) repository at https://www.ncbi.nlm.nih.gov/sra/PRJNA612599, reference number PRJNA612599. The clinical datasets from the CORDIOPREV study used during and/or analyzed during the current study are available from the corresponding authors upon reasonable request.

## Supporting information

Supporting Information

Supporting Information

Supporting Information

Supporting Information

Supporting Information

Supporting Information

Supporting Information

Supporting Information

Supporting Information
