# Supplementary material for: A microbiota‐based predictive model for type 2 diabetes remission induced by dietary intervention: From the CORDIOPREV study
Source: Clin Transl Med. 2021 Apr 6;11(4):e326. doi: 10.1002/ctm2.326 (PMC8023646; doi:10.1002/ctm2.326)
Supplement: Supplementary file 1 — Supporting Information [file CTM2-11-e326-s008.pdf]

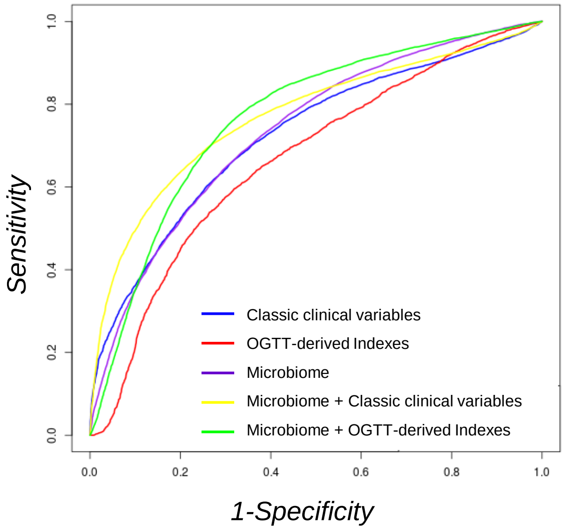

	ROC	Sensitivity	Specificity	Accuracy	Kappa coefficient
			Mean ± SD		
Classic clinical variables	0.753±0.150	0.74±0.17	0.66±0.23	0.71±0.13	0.40±0.27
OGGT-derived indexes	0.682±0.156	0.65±0.19	0.64±0.20	0.65±0.12	0.29±0.23
Microbiome	0.762±0.148	0.72±0.17	0.64±0.21	0.69±0.14	0.36±0.28
Microbiome + Classic cinical variables	0.798±0.147	0.78±0.17	0.68±0.21	0.74±0.13	0.46±0.28
Microbiome + OGGT-derived indexes	0.795±0.148	0.77±0.16	0.68±0.22	0.73±0.14	0.44±0.28

Figure S1. Random forest predictive models for diet consumption-induced type 2 diabetes remission adding smoking variable. Data are mean±SD and ROC curves average of the predictive models using cross-validation method. Classic variables (clinical parameters known as risk factors for type 2 diabetes development: gender, age, body mass index, HDL, triglycerides, HbA1c, physical activity, dietary consumption of fruits and vegetables, use of antihypertensive medication, smoking and family history of diabetes); oral glucose tolerance test (OGTT)-derived indexes: homeostatic model assessment of insulin resistance, disposition index, hepatic insulin resistance index, insulin sensitivity index, muscle insulin sensitivity index and insulinogenic index. The models were adjusted by diet and intensity of statin treatment including these variables in all the models. AUC, area under the ROC curve.
