# Supplementary material for: A microbiota‐based predictive model for type 2 diabetes remission induced by dietary intervention: From the CORDIOPREV study
Source: Clin Transl Med. 2021 Apr 6;11(4):e326. doi: 10.1002/ctm2.326 (PMC8023646; doi:10.1002/ctm2.326)
Supplement: Supplementary file 2 — Supporting Information [file CTM2-11-e326-s007.pdf]

Table S1. Change of anthropometric characteristics between baseline and after five years of dietary intervention in Responders and non-Responders groups.

	Responders (n=73)	Non-Responders (n=107)	*p-value*
Body mass index (Kg/m^2)	−0.68 ± 0.20	−0.35 ± 0.21	0.286
Waist circumference (cm)	0.20 ± 0.70	0.29 ± 0.78	0.935
Weight (kg)	−1.74 ± 0.55	−0.98 ± 0.59	0.370

Our study was conducted in 183 newly-diagnosed type 2 diabetes patients, 110 from which had available feces samples and had not received antibiotic treatment within three months before sample collection. Data are mean±SEM. One-Way ANOVA *p*-values. Significant differences ($p < 0.05$). *= The delta change was calculated with the data of the patients who had continued in the study at five years of follow-up (7 patients died before the first year of the study and 3 died between the second year of the intervention).
