# Supplementary material for: A microbiota‐based predictive model for type 2 diabetes remission induced by dietary intervention: From the CORDIOPREV study
Source: Clin Transl Med. 2021 Apr 6;11(4):e326. doi: 10.1002/ctm2.326 (PMC8023646; doi:10.1002/ctm2.326)
Supplement: Supplementary file 3 — Supporting Information [file CTM2-11-e326-s001.pdf]

Table S2. Association of T2DM remission with the type of diet consumed.

	Non-Responders	Responders	*p-value*
**LF diet**†	63 (61.2)	40 (38.8)	0.741
**Med diet**‡	47 (58.8)	33 (41.2)	

Our study was conducted in 183 newly-diagnosed type 2 diabetes patients, 110 from which had available feces samples and had not received antibiotic treatment within three months before sample collection. Number of patients (percentage of patients according to diet group). Chi-square test p-value. Significant differences ($p < 0.05$). †LF diet: Low-fat diet; ‡Med diet: Mediterranean diet.
