# Supplementary material for: A microbiota‐based predictive model for type 2 diabetes remission induced by dietary intervention: From the CORDIOPREV study
Source: Clin Transl Med. 2021 Apr 6;11(4):e326. doi: 10.1002/ctm2.326 (PMC8023646; doi:10.1002/ctm2.326)
Supplement: Supplementary file 4 — Supporting Information [file CTM2-11-e326-s005.pdf]

Table S3. Analysis of α-diversity.

**Diversity**	**Responders**	**Non-Responders**	***p-value***
Chao1	199.6±5.8	196.7±3.8	0.665
Simpson	0.933±0.002	0.935±0.001	0.380
Shannon	3.16±0.03	3.18±0.02	0.652

Data are mean±SEM. Responders group: patients who reverted from T2DM after 5 years of dietary intervention follow-up. Non-Responders group: patients who remained with T2DM after 5 years of follow-up. Significant differences ($p < 0.05$) by Mann-Whitney U test.
