# Supplementary material for: A microbiota‐based predictive model for type 2 diabetes remission induced by dietary intervention: From the CORDIOPREV study
Source: Clin Transl Med. 2021 Apr 6;11(4):e326. doi: 10.1002/ctm2.326 (PMC8023646; doi:10.1002/ctm2.326)
Supplement: Supplementary file 5 — Supporting Information [file CTM2-11-e326-s003.pdf]

Table S4. Baseline characteristics of the groups according to tertiles of the microbiome-based response prediction score.

	High Response Prediction Score (n=37)	Intermediate Response Prediction Score (n=37)	Low Response Prediction Score (n=36)	*p* value
Men/Women	35/2	28/9	29/7	0.074
Age (years)	55.9±1.4	60.6±1.7	59.6±1.5	0.081
Weight (kg)	86.8±2.2	83.4±2.6	81.9±2.2	0.339
Body mass index (kg/m^2)	31.7±0.6	30.5±0.8	30.0±0.7	0.201
Waist circumference (cm)	106±2	105±2	102±1	0.291
Triglycerides (mmol/L)	1.66±0.13	1.73±0.16	1.60±0.11	0.781
Total-cholesterol (mmol/L)	4.18±0.10	4.25±0.11	4.38±0.19	0.571
HDL-cholesterol (mmol/L)	1.03±0.03	1.06±0.04	1.13±0.06	0.319
LDL-cholesterol (mmol/L)	2.36±0.10	2.39±0.09	2.26±0.15	0.704
C-reactive protein (nmol/L)	30.5±5	37.9±7	34.9±8	0.726
HbA1c (mmol/mol)	49.1±1.2	50.4±1.5	49.5±2.2	0.854
HbA1c (%)	6.64±0.11	6.76±0.13	6.68±0.20	0.854
Glucose (mmol/L)	6.06±0.17	5.92±0.21	6.38±0.30	0.358
Insulin (nmol/L)	102±20	75.5±8.7	71.1±7.9	0.226
HOMA-IR	5.05±0.75	3.83±0.31	4.42±0.80	0.416
Insulin sensitivity index	2.55±0.27	2.65±0.19	2.87±0.28	0.664
Insulinogenic index	1.02±0.38	1.01±0.31	0.47±0.09	0.314
Hepatic insulin resistance index	2077±300	1569±123	1790±323	0.392
Muscle Insulin sensitivity index (x10^2)	2.03±0.40	2.31±0.44	1.77±0.41	0.660
Disposition Index	0.57±0.06[a,b]	0.61±0.05[a]	0.43±0.04[b]	0.040

Our study was conducted in 183 newly-diagnosed type 2 diabetes patients, 110 from which had available feces samples and had not received antibiotic treatment within three months before sample collection. Data are mean±SEM. Groups were determined categorizing patients by ascending tertiles of the microbiome-based response prediction score value: T1, Low-response prediction score; T2, Intermediate-response prediction score; T3, High-response prediction score. Variables were calculated by One-way ANOVA. Gender p value: Chi square analysis. Different letters indicate significant differences (p<0.05) between groups in the post hoc Bonferroni's multiple comparison tests.
