# Supplementary material for: A microbiota‐based predictive model for type 2 diabetes remission induced by dietary intervention: From the CORDIOPREV study
Source: Clin Transl Med. 2021 Apr 6;11(4):e326. doi: 10.1002/ctm2.326 (PMC8023646; doi:10.1002/ctm2.326)
Supplement: Supplementary file 6 — Supporting Information [file CTM2-11-e326-s009.pdf]

Table S5. Analysis of serum C-reactive protein levels according to Responders and Non-Responders groups and changes between baseline and after five years of dietary intervention.

	**Responders (n=73)**	**Non-Responders (n=110)**	***p value* of groups***	**Responders† (n=44)**	**Non-Responders† (n=66)**	***p value* of groups***
**C-reactive Protein (nmol/L)**						
Baseline	37.1±5.3	33.5±3.6	0.558	37.8±6.8	32.2±4.5	0.480
5 years	32.0±7.3	46.4±9.6	0.268	31.7±11.7	35.3±5.8	0.763
p value# of time	0.200	0.151	----	0.351	0.152	----
Delta-change	-8.9±6.8	13.9±9.6	0.077	-9.6±10.1	7.3±5.0	0.105

Our study was conducted in 183 newly-diagnosed type 2 diabetes patients, 110 from which had available feces samples and had not received antibiotic treatment within three months before sample collection. Data are mean±SEM. Responders group: patients who reverted from type 2 diabetes after 5 years of dietary intervention follow-up. Non-Responders group: patients who remained with type 2 diabetes after 5 years of follow-up. Responders†: patients who reverted from type 2 diabetes after 5 years of dietary intervention follow-up to which we have availability of fecal sample. Non-Responders†: patients who remained with type 2 diabetes after 5 years of follow-up to which we have availability of fecal sample. *Signifcant diference ($p < 0.05$) between the Responder and Non-Responders group, analyzed using One-way ANOVA. #Signifcant diference ($p < 0.05$) between baseline and after 5 years of follow-up in each group, analyzed using a paired t test.
