# Supplementary material for: A microbiota‐based predictive model for type 2 diabetes remission induced by dietary intervention: From the CORDIOPREV study
Source: Clin Transl Med. 2021 Apr 6;11(4):e326. doi: 10.1002/ctm2.326 (PMC8023646; doi:10.1002/ctm2.326)
Supplement: Supplementary file 7 — Supporting Information [file CTM2-11-e326-s004.pdf]

**Table S6. Analysis of antibiotics consumption during the dietary intervention follow-up according to Responders and Non-Responders groups.**

| | **Responders (n=73)** | **Non-Responders (n=110)** | ***p value*** | **Responders† (n=44)** | **Non-Responders† (n=66)** | ***p value*** |
|---|---|---|---|---|---|---|
| *Antibiotics Consumption during the follow-up (%)* | 79.5 | 80.9 | 0.808 | 72.7 | 78.8 | 0.464 |
| *Nº of times* | 6.29±0.68 | 6.09±0.73 | 0.848 | 5.72±0.84 | 6.23±1.02 | 0.726 |

Our study was conducted in 183 newly-diagnosed type 2 diabetes patients, 110 from which had available feces samples and had not received antibiotic treatment within three months before sample collection. Responders group: patients who reverted from type 2 diabetes after 5 years of dietary intervention follow-up. Non-Responders group: patients who remained with type 2 diabetes after 5 years of follow-up. Responders†: patients who reverted from type 2 diabetes after 5 years of dietary intervention follow-up to which we have availability of fecal sample. Non-Responders†: patients who remained with type 2 diabetes after 5 years of follow-up to which we have availability of fecal sample. The percentage of patients treated with antibiotics during the follow-up was analyzed by chi-square. Nº of times that patients were treated with antibiotics during the follow-up was analyzed using One-way ANOVA (Data are mean±SEM). Signifcant diference ($p < 0.05$).
