# Supplementary material for: A microbiota‐based predictive model for type 2 diabetes remission induced by dietary intervention: From the CORDIOPREV study
Source: Clin Transl Med. 2021 Apr 6;11(4):e326. doi: 10.1002/ctm2.326 (PMC8023646; doi:10.1002/ctm2.326)
Supplement: Supplementary file 8 — Supporting Information [file CTM2-11-e326-s002.pdf]

Table S7. Adherence to diets by Responders and Non-Responders participants before and after dietary intervention.

Baseline	**Responders**	**Non-Responders**	***p value***	**Responders**[†]	**Non-Responders**[†]	***p value***
LF adherence score	3.88±0.2	3.78±0.1	0.653	3.68±0.2	3.74±0.2	0.822
Med adherence score	9.01±0.2	8.62±0.2	0.136	8.70±0.3	8.55±0.2	0.650
*5-years**	**Responders**	**Non-Responders**	***p value***	**Responders**[†]	**Non-Responders**[†]	***p value***
LF adherence score	7.39±0.3[a]	7.24±0.2[b]	0.646	6.86±0.4[a]	7.42±0.3[b]	0.209
Med adherence score	11.53±0.3[a]	11.33±0.2[b]	0.583	9.47±0.4	9.32±0.3[b]	0.763

Our study was conducted in 183 newly-diagnosed type 2 diabetes patients, 110 from which had available feces samples and had not received antibiotic treatment within three months before sample collection. Data are mean±SEM. Responders group: patients who reverted from type 2 diabetes after 5 years of dietary intervention follow-up. Non-Responders group: patients who remained with type 2 diabetes after 5 years of follow-up. Responders[†]: patients who reverted from type 2 diabetes after 5 years of dietary intervention follow-up to which we have availability of fecal sample. Non-Responders[†]: patients who remained with type 2 diabetes after 5 years of follow-up to which we have availability of fecal sample. One-Way ANOVA p-values ($P<0.05$). ANOVA for repeated measures: [a] $P<0.05$ between baseline and five years in Responders; [b] $P<0.05$ between baseline and five years in Non-Responders. *Data at year five correspond to the patients randomized to each diet.
