# Supplementary material for: A microbiota‐based predictive model for type 2 diabetes remission induced by dietary intervention: From the CORDIOPREV study
Source: Clin Transl Med. 2021 Apr 6;11(4):e326. doi: 10.1002/ctm2.326 (PMC8023646; doi:10.1002/ctm2.326)
Supplement: Supplementary file 9 — Supporting Information [file CTM2-11-e326-s006.pdf]

Table S8. Medical treatment during the dietary intervention according to Responders and Non-Responders groups.

Treatment during the follow-up (%)	Responders	Non-Responders	*p value*	Responders†	Non-Responders†	*p value*
Anti-Aggregates	94.6	94.0	0.882	92.1	96.2	0.395
Alpha-blockers	8.9	11.9	0.577	7.9	11.3	0.589
Beta-blockers	76.8	81.0	0.551	73.7	83.0	0.280
ACE-inhibitors	45.2	34.5	0.147	57.9	45.3	0.235
Angiotensin-II receptor blockers (ARBs)	26.8	44.0	0.038	28.9	41.5	0.219
Calcium Antagonists	26.8	22.6	0.577	25.0	19.7	0.509
Statins	94.6	97.6	0.353	92.1	96.2	0.395
Proton pump inhibitors	76.8	82.1	0.438	78.9	88.7	0.204
Opioids	16.1	19.0	0.652	21.1	18.9	0.796
SSRI antidepressants	8.9	10.7	0.730	7.9	7.5	0.951
Tricyclic antidepressants	0.0	1.2	0.413	0.0	1.9	0.395
Vitamin D (cholecalciferol)	1.8	3.6	0.534	2.6	1.9	0.811
Laxatives	5.4	0.0	0.032	5.3	0.0	0.091
Metformin	0.0	57.1	<0.001	0.0	60.4	<0.001
Other antidiabetic drugs	0.0	11.9	0.007	0.0	15.1	0.012

Our study was conducted in 183 newly-diagnosed type 2 diabetes patients, 110 from which had available feces samples and had not received antibiotic treatment within three months before sample collection. Data are percentage of patients who consumed each type of treatment were. Responders group: patients who reverted from type 2 diabetes after the dietary intervention follow-up. Non-Responders group: patients who remained with type 2 diabetes after the follow-up period. Responders†: patients who reverted from type 2 diabetes after the dietary intervention follow-up to which we have availability of fecal sample. Non-Responders†: patients who remained with type 2 diabetes after the follow-up to which we have availability of fecal sample. SSRI: selective serotonin reuptake inhibitor. *p* values were calculated by Chi square analysis ($p<0.05$).
